# Sulfiredoxin May Promote Cervical Cancer Metastasis via Wnt/β-Catenin Signaling Pathway

**DOI:** 10.3390/ijms18050917

**Published:** 2017-04-27

**Authors:** Kangyun Lan, Yuni Zhao, Yue Fan, Binbin Ma, Shanshan Yang, Qin Liu, Hua Linghu, Hui Wang

**Affiliations:** Department of Obstetrics and Gynecology, The First Affiliated Hospital of Chongqing Medical University, Chongqing 400016, China; kangyun_lan@126.com (K.L.); tutoo250@sina.com (Y.Z.); lemon.1993@163.com (Y.F.); 13220309803@163.com (B.M.); yss133yss133@126.com (S.Y.); liudier771@126.com (Q.L.); linghu_hua@126.com (H.L.)

**Keywords:** sulfiredoxin, cervical cancer, metastasis, Wnt/β-catenin signaling

## Abstract

The abnormal elevation of sulfiredoxin (Srx/SRXN1)—an antioxidant enzyme whose main function is to protect against oxidative stress—has been shown to be closely correlated with the progression of several types of cancer, including human cervical cancer. However, the molecular mechanism by which Srx promotes tumor progression, especially cancer metastasis in cervical cancer, has not been elucidated. Here, we show that Srx expression gradually increases during the progression of human cervical cancer and its expression level is closely correlated with lymph node metastasis. Our study also reveals a significant positive correlation between the expression of Srx and β-catenin in cervical cancer tissues. Loss-of-function studies demonstrate that Srx knockdown using a lentiviral vector-mediated specific shRNA decreases the migration and invasion capacity in HeLa (human papilloma virus 18 type cervical cancer cell line) and SiHa SiHa (cervical squamous cancer cell line). Notably, the exact opposite effects were observed in gain-of-function experiments in C-33A cells. Mechanistically, downregulation or upregulation of Srx leads to an altered expression of proteins associated with the Wnt/β-catenin signaling pathway. Furthermore, blockage of the Wnt/β-catenin signaling pathway contributed to attenuated Srx expression and resulted in significant inhibition of cell migration and invasion in cervical cancer cell lines. Combined, Srx might be an oncoprotein in cervical cancer, playing critical roles in activating the Wnt/β-catenin signaling pathway; it may therefore be a therapeutic target for cervical cancer.

## 1. Introduction

Cervical cancer is the second leading cause of death among young women aged 19–39 years [1], and the fourth leading cause of mortality in females worldwide [2]. Many patients with cervical carcinoma metastasis previously treated with surgery or chemotherapy will develop recurrent disease which seriously affects the quality of life of patients [3]. Thus, it is critical to elucidate the molecular and biologic mechanisms in the development of cervical tumor and develop better therapeutic strategies in cervical cancer.

Sulfiredoxin (Srx) is a novel discovered antioxidant enzyme [4,5], which was initially identified in yeast. The main role of Srx is to reduce its downstream target gene hyperoxidized peroxiredoxin (Prx; a member of antioxidant protein) back to active peroxidases in the presence of ATP [6,7,8], and then counteract the excessive reactive oxygen species (ROS) to protect the host organism from oxidative damages [9]. However, this property of Srx becomes a damaging effect to host cells when it starts protecting the survival of cancer cells [10,11]. As per published literature and the data from the microarray database, Srx is altered in multiple types of cancer and plays critical roles in carcinogenesis by modulating cell signal transduction involved in cell proliferation, migration and metastasis [10]. It has been reported that Srx was upregulated in several human cancers, including colorectal cancer [12], skin malignancies [13], lung cancer [14] and human cervical cancer [15], suggesting the potential role of Srx in tumor. As we mentioned above, Srx is associated with cancer metastasis. For example, Srx can modulate cancer cell motility via redox sensitive interaction with non-muscle myosin IIA (NMIIA) and S100A4 [16], and promotes colorectal cancer cell adhesion and migration through a mechanism of enhancing EGFR (epidermal growth factor receptor) signaling [12]. These studies indicate that Srx plays a critical role in cancer progression and metastasis. However, the complicated function and molecular mechanism of Srx in cervical cancer metastasis has remained largely undiscovered.

Wnt/β-catenin signaling, which activates β-catenin to initiate the transcription of its downstream target genes, has been reported to be associated with carcinogenesis and progression in cervical cancer [17,18,19]. A study showed that Prx, a target gene of Srx [20], has been implicated in the regulation of the Wnt/β-catenin signaling pathway. For example, knockdown of Prx inhibits the growth of colorectal cells by downregulating the Wnt/β-catenin signaling [21]. Our previously study revealed a significant positive correlation between the expression of Srx and E-catenin (an upstream molecule of the Wnt/β-catenin signaling pathway) in cervical cancer tissues [15].

In light of these observation, we hypothesize that Srx may be an important molecule in human cervical cancer development and progression. We assessed Srx expression in human cervical tissue specimens, including normal cervical squamous cell epithelium tissues (NC), cervical intraepithelial neoplasia tissues (CIN) and human cervical cancer tissues; investigated the biological function of Srx in cervical cancer; and examined whether these effects are mediated by the Wnt/β-catenin signaling pathway.

## 2. Results

Srx and β-catenin are overexpressed and correlated with metastasis in cervical cancer, and the expression of the two proteins is positively correlated.

**Figure 1:**
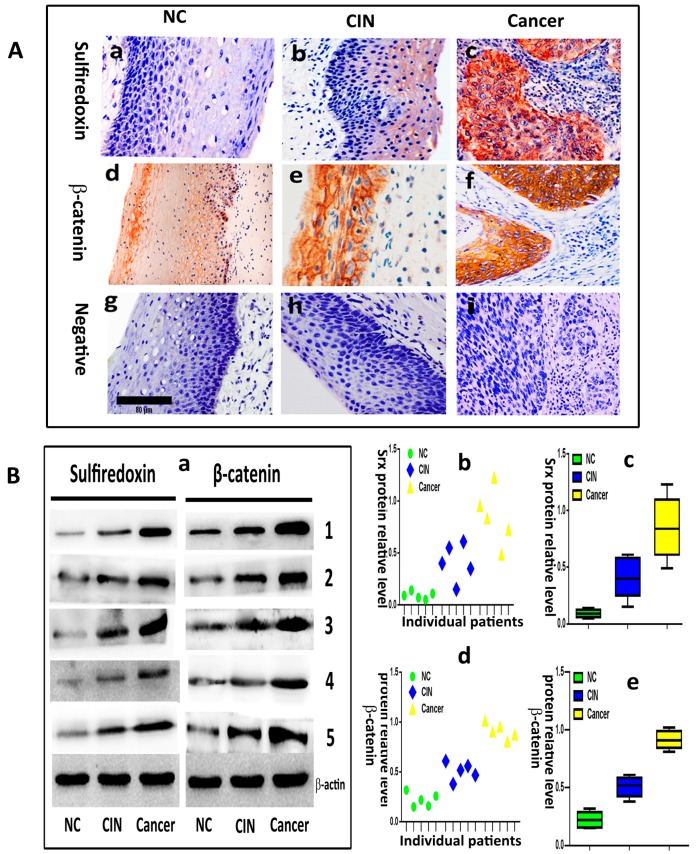
Sulfiredoxin (Srx) and β-catenin are highly expressed in human cervical cancer tissues. (**A**) Immunohistochemical staining to detect Srx and β-catenin. (**a**) Normal cervical (NC) tissues without Srx expression; (**b**) Cervical intraepithelial neoplasia (CIN) with mild expression of Srx; (**c**) Strong positive staining for Srx was observed in cervical cancer tissues; (**d**) Cytomembrane expression of β-catenin in NC tissues; (**e**) Partial defect of β-catenin in cytomembrane from CIN; (**f**) Cytoplasmic and nuclear strong positive staining of β-catenin in cervical cancer tissues; (**g**–**i**) No measurable staining of PBS (phosphate belanced solution )was used as a negative control; (**a**–**i**) Original magnification 200×. All the images in the Figure 2B have the same scale bar; (**B**) The protein expression of Srx and β-catenin in NC, CIN and cervical cancer tissues was assayed by Western blotting. (**a**) Western blotting showing the expression of Srx in NC, CIN and cervical cancer tissues; (**b**) Dot plot of individual patients and their corresponding Srx protein relative level; (**c**) Box plot shows median, lower and upper quartiles, and minimum and maximum values of Srx protein relative level for NC cases, CIN cases and cervical cancer cases; (**d**) Dot plot of individual patients and their corresponding β-catenin protein relative level; (**e**) Box plot shows median, lower and upper quartiles, and minimum and maximum of β-catenin protein relative level for NC cases, CIN cases and cervical cancer cases. Srx and β-catenin expression was significantly increased in cervical cancer tissues. (*p* < 0.05, NC vs. CIN; *p* < 0.01, NC vs. Cancer). The expression of β-actin was used as a loading control.

We evaluated the expression of Srx and β-catenin in 20 normal cervical samples (NC), 30 cervical intraepithelial neoplasia (CIN) and 90 human cervical cancer tissues by immunohistochemistry. The results showed that Srx was predominantly localized in the cytoplasm of cervical cancer tissues (Figure 1(Ac)), and was rarely found in NC tissues (Figure 1(Aa)). Based on the expression scores, the percentage of Srx positive expression samples increased gradually from 15% in NC, to 46.7% in CIN to 73.3% in cervical cancer (Table 1). β-catenin was located in the membrane of the cell in NC (Figure 1(Ad)). However, its location transfers to the cytoplasm and nucleus in cervical cancer (Figure 1(Af)). The positive rate of β-catenin in cervical cancer (78.9%) and CIN (53.3%) was higher than in NC (20%) (Table 1). The differences in the positive expression rate of Srx or β-catenin in NC, CIN, and cervical cancer groups were statistically significant (*p* < 0.05). Then, we evaluated the expression of Srx and β-catenin in five NC, five CIN and five cervical cancer tissues by Western blotting, and the results showed that the two proteins significantly upregulated in both NC and CIN tissues, compared to the NC group (*p* < 0.05; Figure 1B). These findings indicated that Srx and β-catenin are highly expressed in human cervical cancer tissues (Figure 1 and Table 1). Next, we assessed the correlation between the expression of the two proteins and the clinicopathological features of cervical cancer, respectively. In these 90 patients with cervical cancer, there were significant associations between Srx expression and lymph node metastasis (*p* < 0.05) or infiltration of the haemal tube (*p* < 0.05), but we did not find a correlation between Srx expression and age, tumor size, degree of histologic differentiation, clinical stage or depth of cancer invasion (*p* > 0.05) (Table 2). The data also revealed that expression of β-catenin was closely associated with lymph node metastasis (*p* < 0.05) (Table 2). Furthermore, Spearman’s rank correlation analysis showed a significant positive correlation between Srx expression and β-catenin expression in human cervical cancer tissues (*r* = 0.365, *p* = 0.000) (Table 3).

### 2.1. Srx Promotes the Migration and Invasion of Cervical Cancer Cells

**Figure 2:**
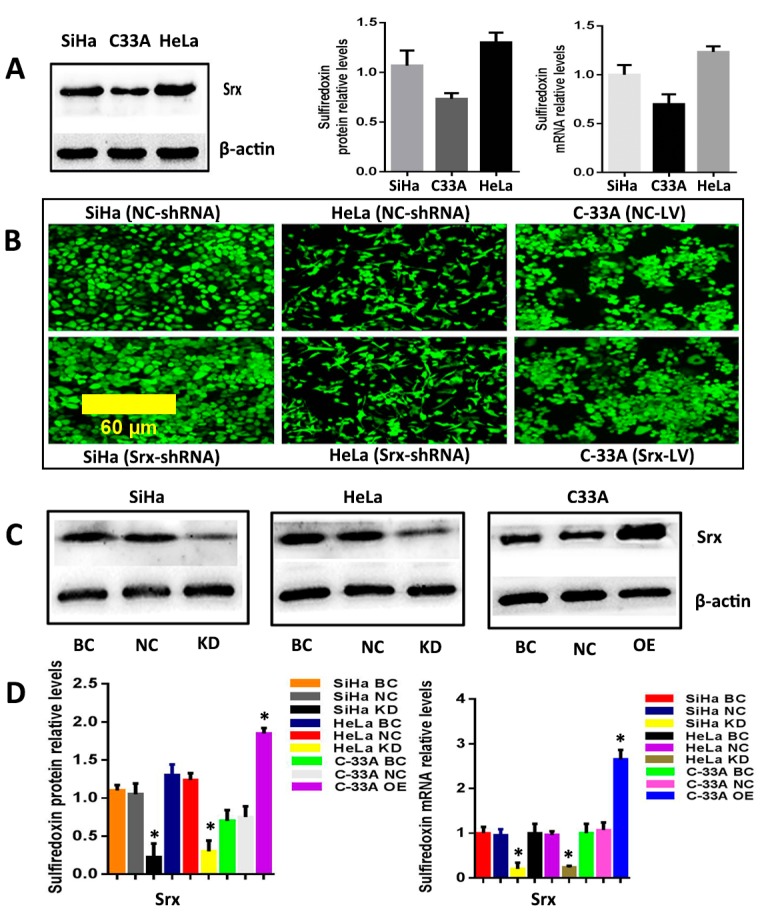
Srx was knocked down by Srx-shRNA and overexpressed by Srx-LV efficiently in cervical cancer cell lines. (**A**) Western blotting (left panel) and qRT-PCR (right panel) were used to detect the expression of Srx in HeLa (human papilloma virus 18 type cervical cancer cell line), SiHa (cervical squamous cancer cell line).and C33A cervical cancer cell lines; (**B**) HeLa and SiHa cells were transduced with lentivirus containing Srx-shRNA, and C33A cells were transduced with lentivirus containing Srx-LV for 72 h, respectively. GFP signals were measured by fluorescence microscopy (100×) and showed that lentiviruses were successfully transduced into cervical cancer cell lines. All the images in the Figure 2B have the same scale bar; (**C**,**D**) Overexpressed and knockdown efficiency of Srx were confirmed by Western blotting (**C** and the left panel of **D**) and qRT-PCR (the right panel of **D**). The NC-LV-transduced cells (NC-LV) and NC-shRNA-transduced cells (NC-shRNA) were used as negative control (NC). Non-transduced cells were used as blank control (BC). The results are expressed as mean ± S.D. * *p* < 0.05.

**Figure 3:**
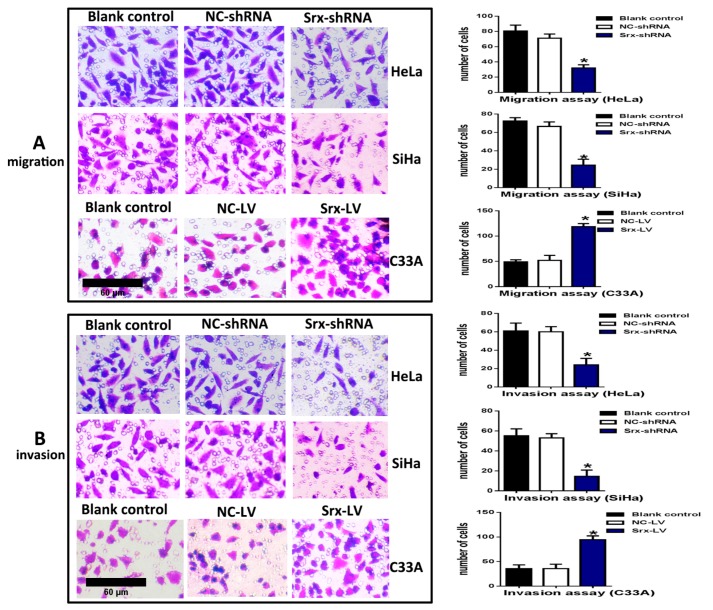
Srx promoted the migration and invasion of cervical cancer cells by the transwell assay. (**A**) The migration of HeLa (upper panels) and SiHa cells (middle panels) was significantly inhibited with Srx knockdown in comparison to the blank control (BC) and NC-shRNA-transduced negative control (NC) group cells. C33A cell (lower panels) migration was markedly increased with Srx overexpression in comparison to the BC and NC-LV-transduced negative control (NC) group cells, as determined by the transwell assay without Matrigel; (**B**) The invasion of HeLa (upper panels) and SiHa cells (middle panels) was significantly inhibited with Srx knockdown in comparison to BC and NC group cells. C33A cell (lower panels) invasion was markedly increased with Srx overexpression in comparison to the BC and NC group cells, as determined by the transwell assay coated with Matrigel. The cells on transwell chambers were fixed, dyed and photographed after culture for 24 h (invasion) or 22 h (migration). All the images in the Figure 2A,B have the same scale bar. The values are expressed as the mean ± SD. (* *p* < 0.05 vs. control).

The correlation between Srx and human cervical cancer metastasis suggested that Srx may play a role in the process of cervical cancer migration and invasion. To test this hypothesis, we subsequently established an effective cell model and performed the transwell assay to measure it. First, we examined the expression of Srx in HeLa, SiHa and C33A cervical cancer cells by Western blotting and qRT-PCR, and results showed that Srx was highly expressed in the HeLa and SiHa cells (Figure 2A). Then, we knocked down Srx in HeLa and SiHa cells by transfecting lentiviruses containing Srx shRNA (Srx-shRNA) (Figure 2B–D), and explored loss-of-function of Srx in human cervical cancer cell lines. The transwell assay showed that cell migration (Figure 3A) (*p* < 0.05) and invasion (Figure 3B) (*p* < 0.05) were significantly reduced with Srx knockdown in HeLa cell lines. The role of Srx knockdown in SiHa cells was similar to that in HeLa cells. Knockdown of Srx inhibited migration (Figure 3A) (*p* < 0.05) and invasion (Figure 3B) (*p* < 0.05) in SiHa cells. Gain-of-function of Srx in C33A cell lines by transfecting lentiviruses containing Srx (Srx-LV) (Figure 2B–D) revealed that overexpression of Srx promoted C33A cell migration (Figure 3A) (*p* < 0.05) and invasion (Figure 3B) (*p* < 0.05).

### 2.2. Silencing or Overexpression of Srx Resulted in Alteration of Proteins Levels Associated with Wnt/β-Catenin Signaling Pathway

**Figure 4:**
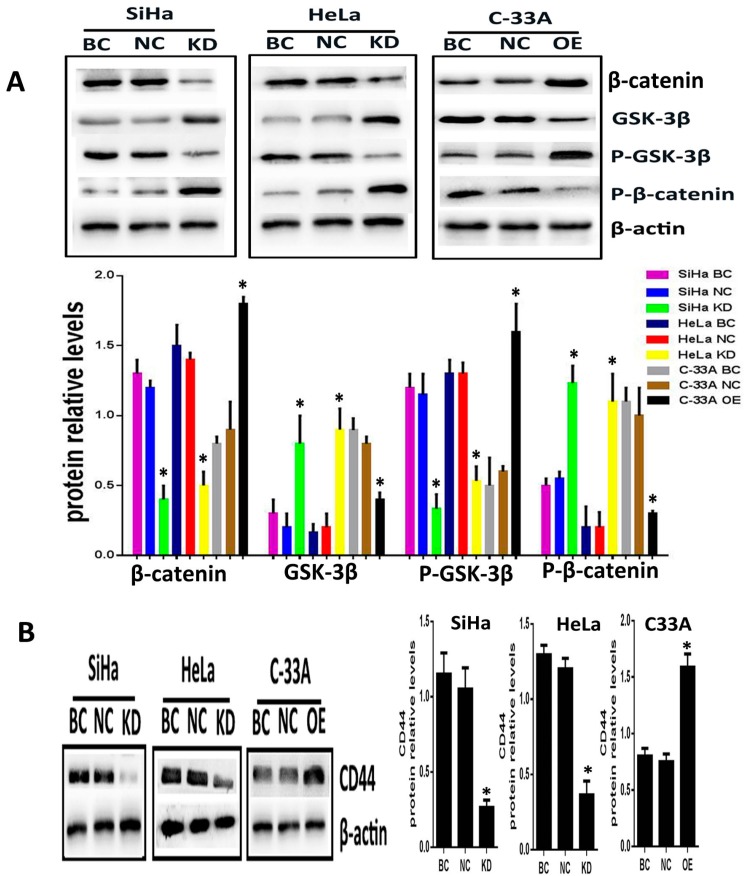
Knockdown of Srx expression inhibits the activation of Wnt/β-catenin signal pathways and upregulation of Srx level promotes the activation of Wnt/β-catenin signal pathways. (**A**) The expression of β-catenin, P-β-catenin, GSK-3β and p-GSK-3β in Srx-depleted HeLa and SiHa cells and Srx-upregulated C33A cells were determined by Western blotting; (**B**) The expression of CD44 (Wnt/β-catenin pathway target genes) in Srx-depleted HeLa and SiHa cells and Srx-upregulated C33A cells were measured by Western blotting. β-Actin was used as the loading controls (* *p* < 0.05 vs. controls).

To explore whether Srx promotes the migration and invasion of cervical cancer cells via Wnt/β-catenin signaling, Srx was silenced in HeLa and SiHa cell lines by lentiviruses containing Srx shRNA, and overexpressed in C33A cell lines by lentiviruses containing Srx-LV. In the present study, with the alteration of Srx expression, we focused on the changing of β-catenin and GSK-3β activity, and the transcriptional activity of target genes. Following over-expression of Srx, total expression of intracellular β-catenin was significantly increased in C33A cells (*p* < 0.05; Figure 4A). In addition, the phosphorylated β-catenin (P-β-catenin), which was generated to break down the Wnt signal, was appreciably decreased with Srx over-expression (*p* < 0.05; Figure 4A). Furthermore, GSK/3β, a critical factor of the Wnt signal, was decreased when Srx was overexpressed in C33A cells, whereas the level of phosphorylated GSK/3b (p-GSK/3b) was notably increased with Srx upregulated in C33A cells (*p* < 0.05; Figure 4A). CD44, a target gene of the Wnt signal, was remarkably increased with Srx over-expression in C33A cells (*p* < 0.05; Figure 4A). Notably, the exact opposite effects were observed in loss-of-function experiments in HeLa and SiHa cells. β-Catenin, p-GSK/3b and CD44 were significantly reduced by knocking down the expression of Srx in HeLa and SiHa cells. We also found that treatment of HeLa and SiHa cells with Srx shRNA significantly increased the expression of GSK-3β and P-β-catenin (*p* < 0.05; Figure 4). These findings indicated that Srx may be involved in the regulation of the Wnt/β-catenin signal pathway in human cervical cancer cells.

### 2.3. The Suppression of Wnt/β-Catenin Signaling Pathway by XAV-939 Inhibits Migration, Invasion and Srx Expression in Cervical Cancer Cell Lines

**Figure 5:**
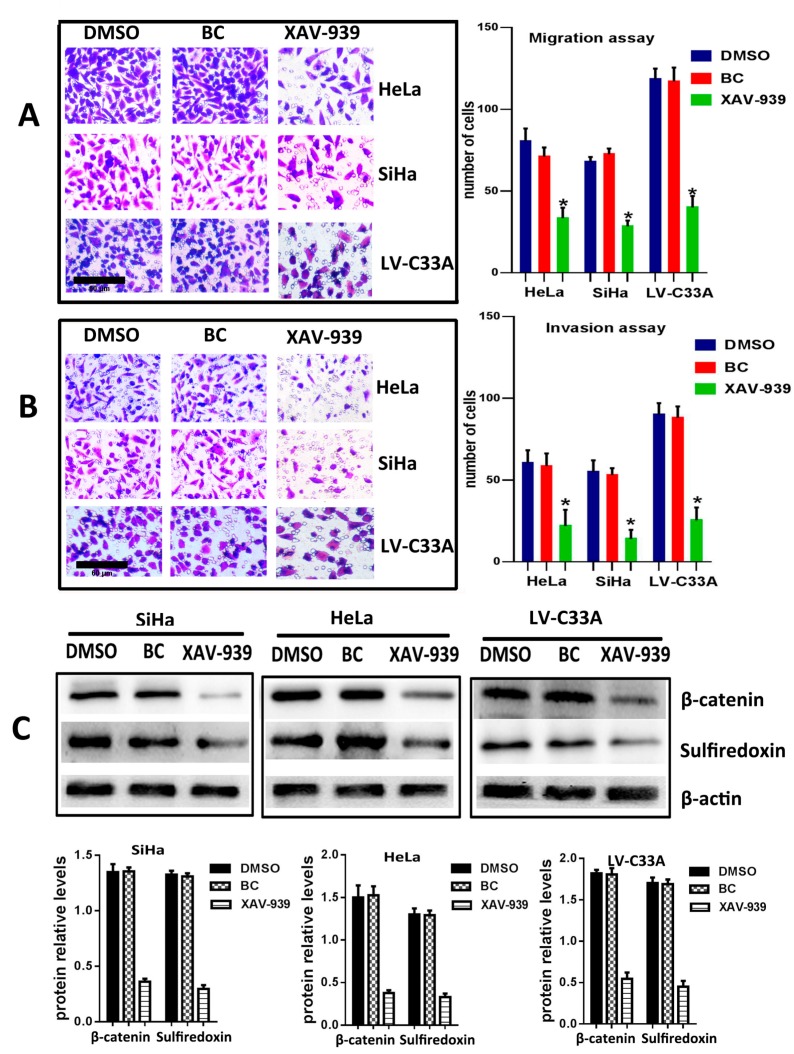
Inhibition of the Wnt/β-catenin signal decreases migration, invasion and Srx expression in cervical cancer cell lines. (**A**) The migration of HeLa, SiHa and LV-C33A cells was markedly inhibited after treatment with XAV-939, an inhibitor of β-catenin, in comparison to the DMSO negative control and blank control (BC), as determined by the transwell assay (* *p* < 0.05 vs. control); (**B**) The effects of XAV-939 on the invasion of HeLa, SiHa and LV-C33A cells were evaluated by the transwell assay, in comparison to the DMSO and BC control (* *p* < 0.05 vs. control); (**C**) Western blotting showed that treatment with XAV-939 in HeLa, SiHa and LV-C33A cells resulted in significant inhibition of β-catenin and Srx expression, in comparison to DMSO and BC control (* *p* < 0.05 vs. control).

To further confirm that the canonical Wnt signaling is the pathway where Srx promotes the migration and invasion of human cervical cancer, XAV-939 (an inhibitor of the canonical Wnt signaling pathway) was used to block Wnt/β-catenin signaling in HeLa, SiHa and Srx-over-expressing C33A (LV-C33A) cell lines. When HeLa, SiHa and LV-C33A cell lines were treated with XAV-939, β-catenin was obviously reduced compared to those in the two cell lines treated with DMSO by Western blotting (*p* < 0.05, Figure 5C). In addition, the inhibition of this pathway caused remarkable suppression of Srx expression (*p* < 0.05, Figure 5C) and highly degreased the migration (*p* < 0.05, Figure 5A) and invasion (*p* < 0.05, Figure 5B) in HeLa, SiHa and LV-C33A cell lines. Taken together, these results suggest that Srx promotes the migration and invasion of cervical cancer cells and may be involved in the activation of the Wnt/β-catenin signaling pathway.

## 3. Discussion

The abnormally elevated expression of Srx was shown to be associated with carcinogenesis in colorectal cancer [22], skin malignancies [23], lung cancer [24], etc. Subsequently, the overexpression of Srx has already been demonstrated to promote cancer metastasis in multiple cancers [10], including those cancers we just mentioned. It is well documented that cancer cells are known to bring about numerous ROS. Thus, it is not difficult to understand that the elevated expression of antioxidant protein such as Srx could be of benefit to cancer cell survival. Furthermore, it is not difficult to understand that cancer treatment may via controlling Srx expression in cancer cells. Based on these observations, our purposes were to elucidate the molecular mechanism by which Srx regulates the metastasis of cervical cancer and establishes the associated signaling mechanisms.

In our present study, the expression of Srx was found to be gradually enhanced from NC tissues to CIN tissues and then to cervical cancer tissues, in agreement with the results of previous studies of cervical cancer [15]. Then, to further explore the function of Srx in cervical carcinogenesis, the correlation between the expression of Srx and clinical pathological features in cervical cancer tissues was analyzed. Furthermore, the results showed that there were significant associations between Srx expression and lymph node metastasis and the infiltration of the haemal tube in cervical cancer tissues. Srx is highly expressed in colorectal cancer cells and is required for colorectal cancer adhesion and migration [12,16], which provides us with evidence that Srx is associated with cancer metastasis. Subsequently, we knocked down Srx in HeLa and SiHa cells by transfecting lentiviruses containing Srx shRNA and upregulated Srx in C33A cell lines by transfecting lentiviruses containing Srx to further confirm that Srx expression might be associated with metastasis in cervical cancer. The results showed that Srx significantly promoted the migration and invasion of cervical cancer cells.

The Wnt/β-catenin signaling pathway, which activates β-catenin to initiate the transcription of its specific downstream target genes [25], has been reported to be associated with numerous cancers [26,27,28,29]. Aberrant activation of the canonical Wnt pathway plays a significant role in human cervical cancer. However, limited data show the correlation between the cancer clinical pathological characteristics and the key molecules such as β-catenin. In our study, we confirmed that β-catenin, indeed, is highly expressed in cervical carcinoma tissues and its expression level was closely associated with lymph node metastasis, which was similar to the findings of other investigators [30]. Based on the published literature, in order to take part in the regulation of serious cancer development, the Wnt/β-catenin signaling pathway does not always stay in the same situation, and is regulated by lots of factors. Here, we suppose that Srx is involved in the regulation of the Wnt/β-catenin signaling pathway. Srx is an antioxidant enzyme that exclusively reduces over-oxidized typical 2-Cys Prx [31], and the reduction of hyperoxidized Prx by Srx can be considered as a rate limiting step in the reduction of hyperoxidized Prx [32]. Individual components of the Srx–Prx axis play critical roles in carcinogenesis by modulating the cell signaling pathway involved in cell migration and metastasis [10]. For example, knockdown of Prx inhibits the growth of colorectal cancer cells via downregulating Wnt/β-catenin signaling [21]. In our study, Spearman’s rank correlation analysis showed a significant positive correlation between Srx expression and β-catenin expression in human cervical cancer tissues. Then, we confirmed that the Wnt/β-catenin pathway was stimulated by Srx in HeLa, SiHa and C33A cells, with activation of CD44—its target genes—resulting in the promotion of invasion and migration in cervical cancer cell lines. All of these studies and evidence offer proof that Srx really is involved in the regulation of the Wnt/β-catenin signaling pathway in human cancer.

## 4. Methods and Materials

### 4.1. Clinical Patient Specimens

A total of 140 cervical specimens, including 20 normal cervical epithelia (NC), 30 cervical intraepithelial neoplasia (CIN) and 90 human cervical cancer tissues, were obtained from the First Affiliated Hospital of Chongqing Medical University (Chongqing, China) from 2010 to 2016. The histological classifications and clinical stages were based on the International Federation of Gynecology and Obstetrics (FIGO) criteria. None of the patients had received chemotherapy, radiotherapy or immunotherapy before specimen collection. All patients voluntarily signed the informed consent before operation. This study protocol was approved by the ethics committee of Chongqing Medical University in 2 March 2015. No. 2015-0302. This study protocol was approved by the ethics committee of Chongqing Medical University.

### 4.2. Cell Lines and Reagents

Human cervical cancer cell lines (HeLa (human papilloma virus 18 type cervical cancer cell line) C33A) were purchased from the Shanghai Cell Bank, Chinese Academy of Sciences (Shanghai, China). Human cervical cancer cell line (SiHa (cervical squamous cancer cell line)) was obtained from Proteintech Group (Wuhan, China). These cell lines were cultured in Dulbecco’s Modified Eagle’s Medium (DMEM; Gibco, Grand Island, NY, USA), respectively supplemented with 10% fetal bovine serum (FBS; Hyclone, Shanghai, China). All cell lines were maintained at 37 °C with 5% CO_2_. XAV-939 was purchased from Sigma-Aldrich (St. Louis, MO, USA), and was added to the cells for 12 h treatment at a final concentration of 5 μM. Culture media were changed after 12 h.

### 4.3. Immunohistochemistry (IHC)

IHC staining was performed using the Immunohistochemical SABC kit (Boster, Wuhan, China) according to the manufacturer’s instructions. The paraffin sections of tissues were deparaffinized as routine. Endogenous peroxidase was removed by 3% H_2_O_2_ for 10 min at 37 °C and antigen was retrieved by citrate buffer (pH 6.0) at 95 °C for 15 min. To prevent non-specific binding, tissues were blocked with 5% bovine serum albumin (BSA) for 20 min at room temperature. Next, tissues were incubated with anti-Srx (1:100; Proteintech; Cat No: 14273-1-AP) and anti-β-catenin (1:100; Proteintech; Cat No: 51067-2-AP) primary rabbit monoclonal antibodies overnight at 4 °C, then goat anti-rabbit secondary antibody for 1 h at 37 °C. Then, the tissues were stained with DAB (diaminobenzidine) dehydrated, fixed and photographed.

Srx and β-catenin expression levels were evaluated based on the staining intensity (0, no staining; 1, light yellow; 2, brown; and 3 dark brown) and the percentage of positive cells (0, <10%; 1, 10–25%; 2, 25–50%; 3, 50–75%; and 4, >75%). Protein staining positivity was calculated by using the following formula: immunoreactivity score (IRS) = intensity score × quantity score. The final score was defined as follows: negative, final score ≤3; weak positive, final score >3; strong positive, final score <6.

### 4.4. Lentiviral Transduction and Establishment of Stable Cell Lines

All lentiviruses, including those containing Srx (Srx-LV) and Srx shRNA (Srx-shRNA) for overexpressing and knocking down Srx, respectively, and control (ctrl-LV) were purchased from GenePharma Co., Ltd. (Shanghai, China). The targeting sequences are Srx-LV (5′-atggggctgcgtgcaggaggaacgctgggcagggccggcgcgggtcggggggcgcccgaggggcccgggccgagcggcggcgcgcagggcggcagcatccactcgggccgcatcgccgcggtgcacaacgtgccgctgagcgtgctcatccggccgctgccgtccgtgttggaccccgccaaggtgcagagcctcgtggacacgatccgggaggacccagacagcgtgccccccatcgatgtcctctggatcaaaggggcccagggaggtgactacttctactcctttgggggctgccaccgctacgcggcctaccagcaactgcagcgagagaccatccccgccaagcttgtccagtccactctctcagacctaagggtgtacctgggagcatccacaccagacttgcagtag-3′) and Srx-shRNA (5′-TCGATGTCCTCTGGATCAA-3′). All the transfection experiments were performed according to the manufacturer’s instructions. Srx-LV was transduced into C33A cells at a multiplicity of infection (MOI) of 60 and Srx-shRNA was transduced into HeLa and SiHa cells at a MOI of 30. Polybrene (GenePharma, Shanghai, China) was added in each well at a final concentration of 5 μg/mL to enhance infection solution. The effects of gene interference on Srx overexpression or silencing were validated using real-time quantitative polymerase chain reaction (RT-qPCR) and Western blotting.

### 4.5. RT-qPCR

Total RNA was extracted using the TRIzol reagent (TaKaRa, Dalian, China) and then reversely transcribed into cDNA using the Prime Script RT Reagent Kit (TakaRa), according to the manufacturer’s instructions. RT-qPCR was performed with the CFX96™ Real-Time System (Bio-Rad, Hercules, California, USA) using SYBR Green (SYBR Premix Ex Taq™ II; TaKaRa) for fluorescent quantification. The following cycling conditions were used for RT-qPCR: pre-denaturation (95 °C, 30 s), denaturation (95 °C, 5 s, 35 cycles), annealing (55–60 °C, 30 s), extension (72 °C, 1 min) and final extension (72 °C, 10 min). The relative mRNA expression was calculated using the 2^−ΔΔ*C*t^ method. The primers were used as follows: Srx (Forward 5′-AAGGTGCAGAGCCTCGTGG-3′ and Reverse 5′-GCTACTGCAAGTCTGGTGTGGA-3′); and β-actin (Forward 5′-CCACGAAACTACCTTCAACTCC-3′ and Reverse 5′-GTGATCTCCTTCTGCATCCTGT-3′).

### 4.6. Western Blot Analysis (WB)

Total Proteins were extracted from tissues and cells by the Protein Extraction Kit (Beyotime, Haimen, China) and the protein concentrations were determined with a BCA (Bicinchoninic acid) protein assay kit (Pierce Biotechnology, Shanghai, China), according to the manufacturer’s instructions. Equal amounts of protein (50 µg) were separated by 10% sodium dodecyl sulfatepolyacrylamide gel electrophoresis (SDS-PAGE) and transferred onto poplvinylidene fluoride (PVDF) membrane. Next, the PVDF membrane was blocked with 5% *w*/*v* non-fat milk for 2 h at room temperature, then incubated with primary antibodies to Srx (1:300; Proteintech), β-catenin (1:500; Cell Signaling Technology, Shanghai, China), GSK/3β (1:500; Cell Signaling Technology), P-β-catenin (1:1000; Cell Signaling Technology), p-GSK/3β (1:1000; Cell Signaling Technology), CD44 (1:1000; Proteintech) and β-actin (1:1000; Abcam, Shanghai, China) at 4 °C with gentle shaking, overnight. After washing with PBS (phosphate belanced solution; PBS) containing 0.05% Tween 20, membranes were incubated with HRP-labeled secondary antibodies (1:2000) for 1.5 h at 37 °C. To calculate protein expression levels, chemiluminescent signals were captured by a chemiluminescence detection system (ChemiDoc™ XRS imager, (Bio-Rad, Hercules, California, USA) and the signal intensity of each PVDF membrane was detected by Fusion software (Vilber Lourmat, Marne-laVallée CEDEX, Shanghai, China).

### 4.7. Cell Migration and Invasion Assay 

For cell migration assay, HeLa, SiHa and C33A cells were re-suspended in serum-free DMEM medium and then seeded into the upper chamber of transwell chambers (Corning Costar, Corning, NY, USA). DMEM medium with 10% FBS was added into the lower chamber as a chemoattractant. After 20 h, the chamber was washed three times with PBS and non-invading cells were removed using a soft cotton swab. Migrated cells on the lower membrane surface were fixed in 4% paraformaldehyde, stained with crystal violet, and photographed at 100× selected in a random manner from five different fields of each sample. Cell invasion assay is basically similar to cell migration assay. The difference is that the transwell chambers used in the cell invasion assay are coated with Matrigel (BD Biosciences, Hercules, CA, USA) and the time of incubation is 24 h. The values for cell migration and invasion were obtained through the mean counting of cells in five fields per membrane. The results are presented as the average of three independent experiments.

### 4.8. Statistical Analyses

All data from these experiments were assessed with statistical software SPSS 20.0 (SPSS, Chicago, IL, USA). The results, not including correlation analysis, from immunohistochemistry were analysis by Pearson’s Chi-Square or Fisher’s exact tests. The correlation between Srx and β-catenin expression was examined by Spearman’s rank correlation. The analysis of variance (ANOVA) was applied to evaluate the significant differences. The data are presented as the mean ± standard deviation. *p* < 0.05 was considered statistically significant. Each experiment was repeated in triplicate.

## 5. Conclusions

In conclusion, our findings indicated that Srx promotes cell invasion and migration in cervical cancer via activating the Wnt/β-catenin signaling pathway. More in-depth mechanistic studies in the future will help to unravel inter weaved behavior of Srx and lead to the development of better therapeutic strategies for cancer prevention and treatment.

## Figures and Tables

**Table 1:** Expression of Srx and β-catenin in different cervical tissues.

Sample	Case	Expression of Srx				Expression of β-Catenin			
		+ (%)	− (%)	χ^2^	*p*	+ (%)	− (%)	χ^2^	*p*
**NC**	20	3(15.0)	17(85.0)			4(20)	16(80.0)		
**CIN**	30	14(46.7)	16(53.3)			16(53.3)	14(46.7)		
**CINI**	5	1(20.0)	4(80.0)			1(20.0)	3(80.0)		
**CINII**	12	5(41.7)	7(58.3)			6(50.0)	6(50.0)		
**CINIII**	13	8(61.5)	5(38.5)			9(69.2)	4(30.8)		
**CSCC**	90	66(73.3)	22(26.7)			71(78.9)	19(21.1)		
**NC vs. CIN**				3.980	0.021			4.253	0.018
**CINvs. Cancer**				7.200	0.013			7.370	0.010
**NC vs. Cancer**				21.386	0.000			23.514	0.000
**NC vs. CIN vs. Cancer**				26.476	0.000			27.037	0.000

CINI (Cervical intra-epithelial-neoplasia1); CINII (Cervical intra-epithelial-neoplasia2); CINIII (Cervical intra-epithelial-neoplasia3). CIN = CINI + CINII + CINIII.

**Table 2:** Correlation between the expression of the two proteins and clinical pathological features of cervical cancer.

Variable	N	Expression of Srx			Expression of β-Catenin		
		+ (%)	− (%)	*p*	+ (%)	− (%)	*p*
Age (year)							
≥45	42	33(78.6)	9(21.4)	0.209	36(85.7)	6(14.3)	0.110
<45	48	33(68.7)	15(31.3)		35(72.9)	13(27.1)	
FIGO stage							
I	70	49(70.0)	21(30.0)	0.146	52(74.3)	18(25.7)	**0.037**
II	20	17(85.0)	3(15.0)		19(95.0)	1(5.0)	
Tumor size (cm)							
≥4	10	6(60.0)	4(40.0)	0.255	6(60.0)	4(40.0)	0.129
<4	80	60(75.0)	20(25.0)		65(81.2)	15(18.8)	
Histologic differentiation							
Well and moderate	63	44(69.8)	19(30.2)	0.190	46(73.0)	17(27.0)	**0.030**
Poor	27	22(81.5)	5(18.5)		25(92.6)	2(7.4)	
Lymph node metastasis							
+	27	24(88.9)	3(11.1)	**0.023**	25(92.6)	2(7.4)	**0.030**
−	63	42(66.7)	21(33.3)		46(73.0)	17(27.0)	
Infiltration of haemal tube							
+	21	19(90.5)	2(9.5)	**0.034**	15(71.4)	6(28.6)	0.252
−	69	47(68.1)	22(31.9)		56(81.2)	13(18.8)	
Depth of cancer invasion							
Light layer	54	36(66.7)	18(33.3)	0.064	41(75.9)	13(24.1)	0.284
Deep layer	36	30(83.3)	6(6.7)		30(83.3)	16(16.7)	

FIGO (International Federation of Gynecology and Obstetrics); “+” means positive expression; “−” means negative expression. Bold values are statistically significant.

**Table 3:** Correlation of Srx and β-catenin expression in cervical cancer tissues.

Expression of β-Catenin	Expression of Srx	
	Positive (*n* = 66)	Negative (*n* = 24)
Positive (*n* = 71)	58	13
Negative (*n* = 19)	8	11
